# OdoriFy: A conglomerate of artificial intelligence–driven prediction engines for olfactory decoding

**DOI:** 10.1016/j.jbc.2021.100956

**Published:** 2021-07-12

**Authors:** Ria Gupta, Aayushi Mittal, Vishesh Agrawal, Sushant Gupta, Krishan Gupta, Rishi Raj Jain, Prakriti Garg, Sanjay Kumar Mohanty, Riya Sogani, Harshit Singh Chhabra, Vishakha Gautam, Tripti Mishra, Debarka Sengupta, Gaurav Ahuja

**Affiliations:** 1 Department of Computational Biology, Indraprastha Institute of Information Technology-Delhi (IIIT-Delhi), New Delhi, India; 2 Department of Computer Science and Engineering, Indraprastha Institute of Information Technology-Delhi (IIIT-Delhi), New Delhi, India; 3 Department of Computer Science and Design, Indraprastha Institute of Information Technology-Delhi (IIIT-Delhi), New Delhi, India; 4 Pathfinder Research and Training Foundation, Greater Noida, Uttar Pradesh, India; 5 Centre for Artificial Intelligence, Indraprastha Institute of Information Technology, New Delhi, India; 6 Institute of Health and Biomedical Innovation, Queensland University of Technology, Brisbane, Queensland, Australia

**Keywords:** Olfactory, chemosensory, machine learning, artificial intelligence, ligand-receptor, computation, computational biology, neuron, chemotaxis, ligand-binding protein, AUC-ROC, area under the curve receiver operating characteristic, BiLSTM, Bidirectional Long Short-Term Memory, CSV, comma-separated value, OR, odorant receptor, PC, principal component, SMILES, Simplified Molecular-Input Line-Entry System

## Abstract

The molecular mechanisms of olfaction, or the sense of smell, are relatively underexplored compared with other sensory systems, primarily because of its underlying molecular complexity and the limited availability of dedicated predictive computational tools. Odorant receptors (ORs) allow the detection and discrimination of a myriad of odorant molecules and therefore mediate the first step of the olfactory signaling cascade. To date, odorant (or agonist) information for the majority of these receptors is still unknown, limiting our understanding of their functional relevance in odor-induced behavioral responses. In this study, we introduce OdoriFy, a Web server featuring powerful deep neural network–based prediction engines. OdoriFy enables (1) identification of odorant molecules for wildtype or mutant human ORs (Odor Finder); (2) classification of user-provided chemicals as odorants/nonodorants (Odorant Predictor); (3) identification of responsive ORs for a query odorant (OR Finder); and (4) interaction validation using Odorant–OR Pair Analysis. In addition, OdoriFy provides the rationale behind every prediction it makes by leveraging explainable artificial intelligence. This module highlights the basis of the prediction of odorants/nonodorants at atomic resolution and for the ORs at amino acid levels. A key distinguishing feature of OdoriFy is that it is built on a comprehensive repertoire of manually curated information of human ORs with their known agonists and nonagonists, making it a highly interactive and resource-enriched Web server. Moreover, comparative analysis of OdoriFy predictions with an alternative structure-based ligand interaction method revealed comparable results. OdoriFy is available freely as a web service at https://odorify.ahujalab.iiitd.edu.in/olfy/.

The sense of smell allows an organism to sense its surroundings by recognizing and processing the information from diverse chemical clues present within the environment. These clues are largely composed of thousands of structurally diverse odorant molecules that mediate vital behavioral responses, such as social communication, identification and quality assessment of food (1), and the recognition of prey and predators (2, 3). Olfactory receptors, the functional units of an olfactory system, are the key drivers of odor perception as they contribute to the first critical step of odorant detection (3, 4, 5, 6, 7). In humans, these specialized classes of receptors are primarily localized in the olfactory epithelia of the olfactory mucosa. Similar to other vertebrates, the human olfactory epithelia contain multiple cell types such as immature and mature olfactory sensory neurons, sustentacular cells, horizontal basal cells, microvillar cells, Bowman's gland cells, globular basal cells, and olfactory ensheathing glia (8, 9, 10, 11). Among these, the olfactory sensory neurons provide direct functional relevance since they harbor the receptors that are responsible for the recognition and discrimination of odorant molecules. Notably, each mature olfactory sensory neuron follows the “one-neuron-one-receptor” rule, that is, each neuron expresses a single functional receptor (9, 12, 13). The axons emanating from the olfactory sensory neurons expressing the same receptor converge to a common target region of the olfactory bulb (14, 15, 16, 17, 18). The mammalian olfactory system is composed of distinct evolutionarily conserved families of chemosensory receptors. These include odorant receptors (ORs), trace amine–associated receptors (8), vomeronasal type 1 and 2 receptors (9), formyl peptide receptors (10), guanylyl cyclases (GUCY2D and GUCY1B2) (11), and the membrane-spanning 4-pass A receptors (12). However, in humans, except for ORs, which constitute the largest gene family, comprising approximately 400 functional OR genes and 600 pseudogenes (6, 19, 20), other gene families collectively contain only a minor fraction of functional receptors (13, 14). For instance, the human genome contains only six functional and single-copy trace amine–associated receptor genes (21, 22). Humans also lack vomeronasal-specific formyl peptide receptors and vomeronasal type 2 receptors (21, 22, 23). Moreover, only five functional vomeronasal type 1 receptors have been reported to date; however, their direct functional importance in pheromone detection is highly debatable (24). This is also supported by the fact that humans lack functional *TRPC2* genes that encode a channel protein, which mediates specific signal transduction in vomeronasal receptors (25). A recent transcriptomics study of human olfactory mucosa revealed the expression of guanylyl cyclases (GUCY2D/GC-D+ or GUCY1B2) as well as membrane-spanning 4-pass A transcripts (26). However, the study lacks companion immunohistological data, leaving uncertainty about the synthesis of associated proteins. Numerous studies suggest that olfactory receptors adopt a combinatorial coding strategy to enable the identification of a myriad of structurally distinct odorant molecules (3, 4, 5, 6, 7). In contrast to other gene families, because of its large number of functional genes, the family of ORs presents a strong case of machine learning–based exploration and identification of their agonists and nonagonists (27). Notably, we use the term agonists for chemicals that are known to interact and activate an OR. Similarly, nonagonists refer to those chemicals that fail to elicit a functional activation response in the receptor–ligand interaction assays. In case, the given chemical activates an OR and possesses an odor percept, we use the term odorants for those chemicals.

Similar to other chemoreceptors, ORs primarily reside on the cilia of the olfactory sensory neurons of the olfactory epithelium. Olfactory signal transduction initiates on the cilia upon odorant or agonist binding to its cognate receptor. Mechanistically, the interaction between an odorant molecule at the binding pocket of an OR triggers conformational changes, resulting in the dissociation of GDP from the Gα subunit (28). These biochemical reactions subsequently lead to the binding of GTP, which further activates adenylyl cyclase III (29). Activated adenylyl cyclase elevates the cellular cAMP levels leading to the opening of cyclic nucleotide–gated channels (29, 30). Channel opening further triggers the depolarization of the ciliary membrane because of the influx of cations. This signal gets further amplified by the opening of calcium-activated chloride channels because of increased intracellular calcium ions (31, 32).

Recent reports utilizing the next-generation sequencing techniques, coupled with high-throughput *in vitro* functional validations, revealed instances of ectopic expression and functionalities of these ORs outside the nasal cavity (33, 34, 35, 36). For instance, under healthy conditions, the OR expression has been reported in multiple organs (37). A handful of ORs expresses in the liver and skeletal muscle, whereas the testis expresses more than 60 ORs (38, 39). Interestingly, in pathological states such as cancer, selective OR expression has been linked to tumor initiation and progression (33, 36, 39, 40, 41). OR51E2, a cancer-associated OR, has shown promising roles as a biomarker potent to guide anticancer therapies (34, 36, 42, 43). The functional experiments involving activation of OR51E2 by nonanoic acid (agonist) in lymph node carcinoma of the prostate cells trigger an antiproliferative response and induction of cellular senescence (44), indicating the therapeutic or diagnostic potential of ORs. A recent bioinformatics analysis utilizing 49 small conditional RNA-Seq datasets revealed the expression of ORs in malignant cells of distinct cancer types at the single-cell resolution (33). Both in the nasal epithelium as well as other tissues, receptor activation is triggered by agonist binding, highlighting the importance of deciphering the entire spectrum of receptor–agonist interactions (36).

While ORs constitute a highly conserved family of functional genes, odorants are rather chemically diversified (45, 46, 47). In general, odorants belong to a class of volatile and structurally diverse chemical compounds with distinct physicochemical properties that impart a preferential binding affinity to their cognate chemosensory receptors (48, 49). However, because of their enormous chemical diversity, efforts are still ongoing to understand the chemical basis of odorant molecules (50). Initial understanding has been that odorants possessing similar functional groups harbor similar perception properties, for example, esters are associated with a fruity smell or a floral smell, whereas thiols induce a rotten smell (51). However, with the increasing number of odorant molecules, it has become apparent that the underlying olfactory mechanisms are more complex than previously anticipated. In recent years, machine learning–based approaches have been implemented in the field of chemosensory research, particularly in predicting the perception response of an odorant or tastant (41, 52, 53, 54, 55). Despite initial interest, the overall adoption of such approaches is still underappreciated. This could be primarily because of the copious amount of effort and expertise required to compile, preprocess, and structurize large volumes of interaction data. To circumvent this gap, we introduce OdoriFy, a comprehensive Web server for deep neural network–based prediction of human odorant–receptor interaction. OdoriFy is built on a comprehensive repertoire of manually curated olfactory information, constituting 5003 odorants, 857 nonodorants, and 6153 interaction pairs (agonist receptor: 679; nonagonist receptor: 5474), making it one of the largest curated data resources to date. In total, OdoriFy contains four prediction engines, that is, Odorant Predictor, Odor Finder, OR Finder, and Odorant–OR Pair Analysis that collectively allow prediction of odorant status, identification of responsive ORs for the given odorant(s), and prediction of putative odorants for user supplemented wildtype or mutant OR protein sequences. In addition to these, OdoriFy also contains modules of explainable artificial intelligence that enable the highlighting of key decision-making structural elements of the predicted odorants or ORs at the atomic or amino acid levels, respectively.

## Results

### OdoriFy: A comprehensive artificial intelligence–driven Web server to explore human olfaction

OdoriFy is an open-source Web server with deep neural network–based prediction models coupled with explainable artificial intelligence functionalities. It is developed with the goal of providing researchers a one-stop destination to decode chemical interactions in the context of olfaction. OdoriFy is capable of identifying agonists for human ORs (Odor Finder), prediction of odorant molecules (Odorant Predictor), identification of responsive ORs for the user-supplied chemicals (OR Finder), and Odorant–OR Pair Analysis. The graphical user interface provides users with a highly automated and hassle-free experience while identifying potential agonists for their OR of interest or validating the presence of odorant properties in the user-supplied chemicals. In the case of Odorant Predictor module, the users can submit query chemicals in the form of SMILES either *via* the upload function (in comma-separated variables) or by directly copying in the input window. To ease user experience in converting their query chemicals into the SMILES format, OdoriFy provides a direct link to the OPSIN Web server (56). Odorant Predictor returns hyperlinked tabular results that include information about the chemical status of being an odorant or nonodorant, its prediction probability, and the highlighted decision determining atoms (interpretable artificial intelligence functionality). The OR Finder prediction engine also takes chemicals as input in the SMILES format. Another companion module, OR Finder, provides two distinct testing methods, that is, rapid and normal testing, where the former is faster but comparatively less accurate as compared with the latter. The output format of OR Finder prediction engine is the same as that of Odorant Predictor, except it also provides the FASTA sequences of the predicted cognate ORs against the user queried chemicals and information on the decision-making amino acids of the OR sequences. Odor Finder allows users to predict the interacting odorants for the user-supplied wildtype or mutated ORs. It allows users to submit the FASTA sequences of their ORs of interest, either using the text window or *via* the upload button. Users can also manipulate the number of top predicted odorants (default set to 5). The prediction output format is similar to that of the aforementioned prediction engines. Last, for the Odorant–OR Pair Analysis, users can submit the matched OR FASTA sequence along with the query chemical, and OdoriFy returns the interaction probabilities along with the other information as discussed previously for the other search engines. Notably, OdoriFy outputs can be downloaded as a single zip file by using the Download Zip button. Of note, all the prediction engines allow users to obtain their results *via* electronic mail.

### OdoriFy supports four distinct olfactory prediction engines

**Figure 1:**
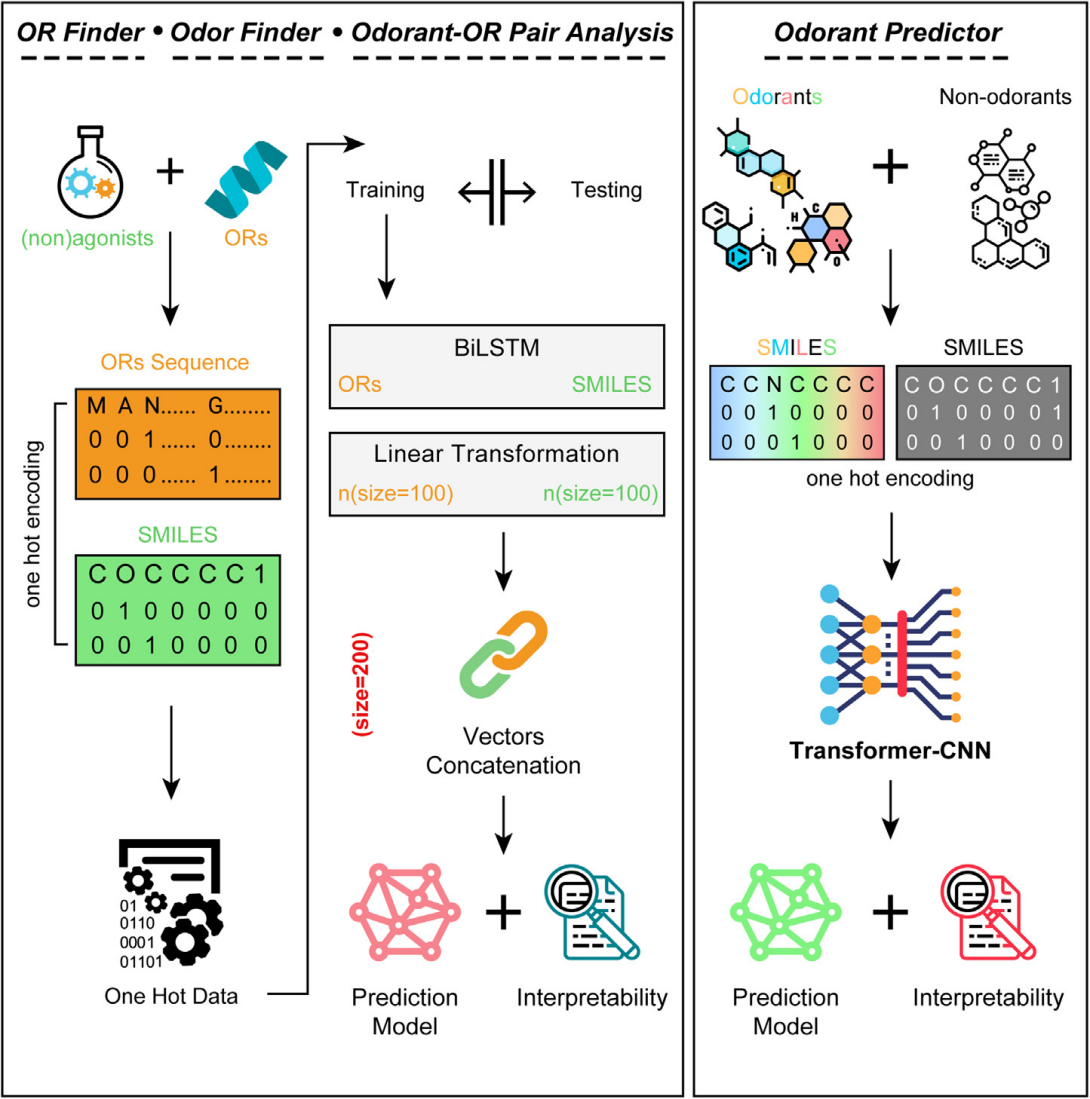
**Deep neural network architectures used to built prediction models for OdoriFy.** A schematic representation of the deep learning architecture used to build models for the OdoriFy prediction engines. The indicated two distinct models provide backend support to four prediction engines, namely Odorant Predictor, Odor Finder, OR Finder, and Odorant–OR Pair Analysis. OR, odorant receptor.

All the supported prediction engines in OdoriFy are substantiated at the backend by two independent deep-learning models (Fig. 1). The first model is exclusive to the Odorant Predictor, whereas the second one supports the remaining three prediction engines. Notably, these distinct models are built using two independent deep neural network architectures, namely Transformer-CNN (57) (Odorant Predictor) and BiLSTM-based methods on One-Hot encoding data (OR Finder, Odor Finder, and Odorant–OR Pair Analysis). By using compiled datasets (refer to Experimental procedures section), we first built the Odorant Predictor, by using the default parameters of the Transformer-CNN workflow (57) (Fig. 1, *right panel*). Moreover, we also utilized its inbuilt functionality for the model interpretation. The second model implemented in OdoriFy is built on BiLSTM-based architecture (Fig. 1, *left panel*). In brief, the input data were randomly split into a training (80% data) and testing (20% data) dataset. One-Hot encoding was performed on the OR amino acid sequences and SMILES of both agonists and nonagonists. BiLSTM is applied to the One-Hot encoded data with a dropout rate of 0.5, and the linear transformation is set to 100 each to prevent model overfitting. Post this step, both the resultant vectors of constant size were concatenated and used for model training. Captum library (58) was used for providing interpretation capabilities at the amino acid (for ORs) and atomic (odorants/nonodorants) levels.

### Odorant Predictor allows identification and explanation of odorants

**Figure 2:**
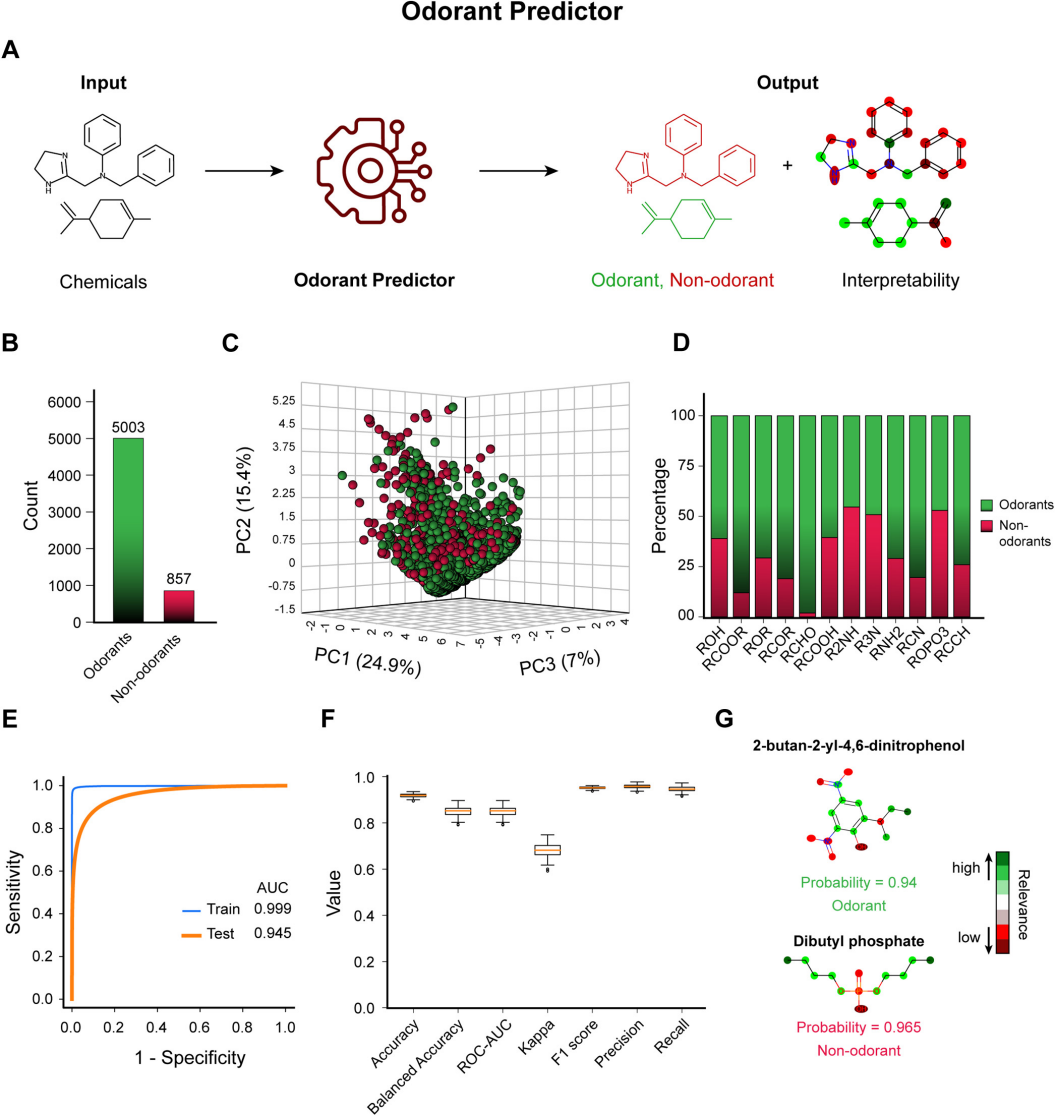
**Odorant Predictor allows rapid classification of user-supplemented chemicals into odorant or nonodorants.***A*, a schematic representation of the critical steps involved in the Odorant Predictor work cycle. Odorant Predictor takes chemical information in SMILES as an input and returns the classification of these chemicals into odorants and nonodorants. *B*, bar plot depicting the number of known odorants and nonodorants in the input dataset used to build the prediction model. *C*, principal component analysis (PCA) on the atom pair fingerprints of known odorants and nonodorants computed using the ChemmineR Bioconductor package. The plot depicts the chemical heterogeneity of odorants and nonodorants along with the three principal components (PC1, PC2, and PC3). *D*, bar graph representing the relative abundance of 12 prominent functional groups (de)enriched in known odorants and nonodorants, collectively describing the relative composition of the functional groups in the input dataset. Of note, the functional group estimation was performed using the ChemmineR package. *E*, AUC plot representing the performance of the best model in classifying the odorants and nonodorants on the training (*blue*) and testing (*orange*) datasets. *F*, box plot representing the distribution of 100 random iterations of the key metrics collectively describing the model performance on the testing (unseen) dataset. The metrics include model accuracy, balanced accuracy, AUC-ROC, Cohen's kappa, F1 score, precision, and recall. *G*, colored chemical structures representing the relative contribution of the highlighted atoms in the classification decision. The *green* and *red colors* represent the highest and lowest contribution thresholds, respectively. The respective odorant status along with prediction probabilities are mentioned below each molecule. AUC-ROC, area under the curve receiver operating characteristic; SMILES, Simplified Molecular-Input Line-Entry System.

In the case of Odorant Predictor, we compiled, refined, and iteratively checked and generated a bonafide list of known odorant and nonodorant molecules from the literature or databases, collectively accounting for 5003 odorant and 857 nonodorant molecules (Fig. 2*B* and Table S1). First, to understand the chemical diversity of odorants and nonodorants in the compiled dataset, we converted them into atom pair fingerprints using the ChemmineR package (59). Atom pair fingerprints describe the properties of atoms and molecules in the form of a numerical vector. Using these atom pair fingerprints as features, we next performed principal component (PC) analysis and visualized the chemical diversity of the bonafide odorants and nonodorants in a three-dimensional space (PC1 *versus* PC2 *versus* PC3) (Fig. 2*C*). Careful interrogation revealed a larger degree of chemical heterogeneity between these molecule classes. Further examination of the input odorants and nonodorants at the functional group level revealed a relatively higher enrichment of certain functional groups such as ethers, esters, and aldehyde among the odorants, which are in line with the previous reports (60, 61) (Fig. 2*D* and Fig. S2*B*). Finally, for the model building, we used Transformer-CNN (57), a computational framework for quantitative structure–activity (property) relationship modeling and interpretation. Transformer-CNN (57) took SMILES as inputs and converted them into embeddings using the One-Hot encoding method and subsequently generated the predictive model (refer to Experimental procedures section). The Odorant Predictor model outputs the AUC-ROC value of 0.945 on the unseen testing dataset, suggesting the remarkable capability of the model to distinguish between the classes (Fig. 2*E*). Other parameters of model performance, such as balanced accuracy, Cohen's kappa, F1 score, precision, and recall, also indicated a high model performance (Fig. 2*F* and Fig. S2*A*). Of note, the model interpretability module of Transformer-CNN (57) provides the putative explanation of the model classification criteria at the atomic level (Fig. 2, *A* and *G*, Figs. S1*A*, and S2, *C* and *D*).

### OdoriFy allows prediction of odorant–OR interaction

**Figure 3:**
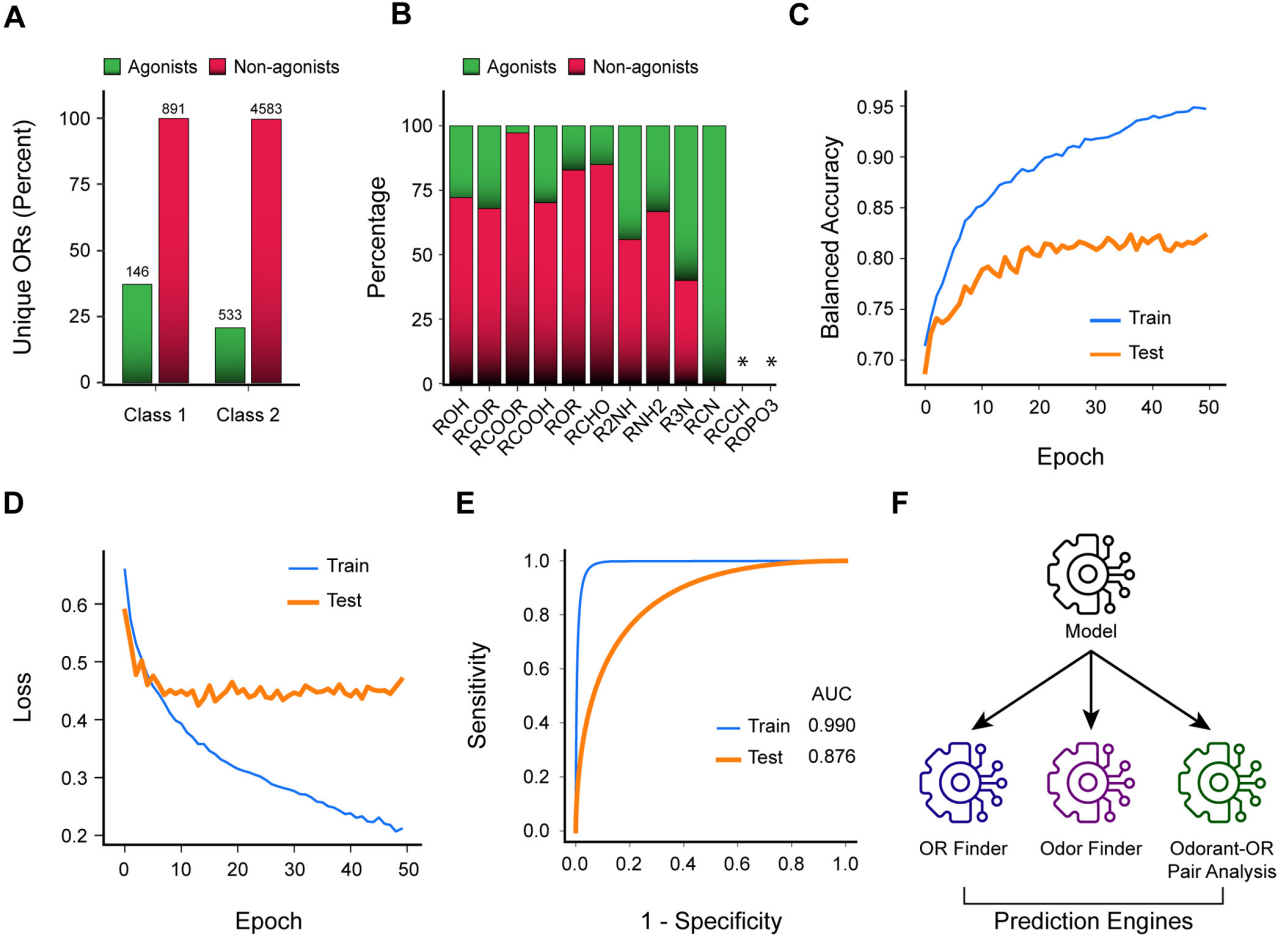
**A unified deep neural network–based prediction model supports Odor Finder, OR Finder, and Odorant–OR Pair Analysis.***A*, bar plot depicting the proportion of human class I and II ORs with the information on known agonists (*green*) and/or nonagonists (*red*). The absolute count of known agonist and nonagonist-OR pairs available for each class is mentioned above each bar in numerics. *B*, bar graph representing the relative abundance of the 12 prominent functional groups enriched in the bonafide agonists and nonagonists, collectively describing the functional group composition of the input dataset. The functional groups were computed using ChemmineR, a Bioconductor package. *Asterisk* indicates the absence of the indicated functional group. *C*, the *line plot* depicts the increase in balanced accuracy over epochs for training and testing datasets where *blue* and *orange colors* represent training and testing data, respectively. *D*, the *line plot* representing the loss across epochs for training and testing datasets. *E*, AUC plot representing the performance of the model in classifying the agonists and nonagonists on the training and test dataset. *F*, schematic representation highlighting a common backend model for the indicated prediction engines. AUC, area under the curve; OR, odorant receptor.

To investigate the potential of deep neural networks in predicting the OR–odorant interactions, we first collated a wealth of information about known agonists/nonagonists for the tested ORs from the public domain (Fig. 3*A* and Table S2). Next, to ensure the quality of the compiled dataset, we carefully removed all the redundant and contradictory entries. We further filtered our dataset by first passing them through the Odorant Predictor engine to preselect only the odorant molecules for the downstream model building (probability cutoff > 0.5) (Fig. S3*A*). Functional group analysis further revealed heterogeneity in the compiled dataset, with comparable enrichment for the individual functional groups between both the agonists and nonagonists. Of note, none of the bonafide agonists or nonagonists in our dataset contained functional groups like RCCH and ROPO3 (Fig. 3*B*). Leveraging the refined dataset, we next built the prediction model using deep neural network techniques involving BiLSTM and linear transformation (Fig. 3, *C* and *D* and Fig. S3*B*). Improving the model performance by hyperparameter tuning revealed the optimal SMILES and OR lengths of 75 and 315, respectively. Moreover, the number of optimal hidden layers was identified to be 50 for both the SMILES and OR sequences. Notably, to resolve the class imbalance issue, we implemented an upsampling approach to augment the number of odorants and applied an iterative process with 100 steps, where at each step, the model was trained on randomly split data and its performance was estimated on the unseen testing data (20%). The best performing model harbored an AUC-ROC value of 0.876 on the unseen testing dataset, suggesting the remarkable capabilities of the model to distinguish between the classes (Fig. 3*E*). Moreover, other parameters for model performance assessment were also within the acceptable range (Fig. S3, *C* and *D*). Finally, we used this as a base model for the OR Finder, Odor Finder, and Odorant–OR Pair Analysis prediction engines (Fig. 3*F*). Taken together, by leveraging one of the largest and highly heterogeneous wealth of known information about human OR–odorants/nonodorants interaction data, coupled with a deep learning–based approach, we built a high-performance prediction model that allows prediction/testing of OR-(non)odorant interaction.

### OdoriFy supports multifacet investigation of odorant–OR interactions

**Figure 4:**
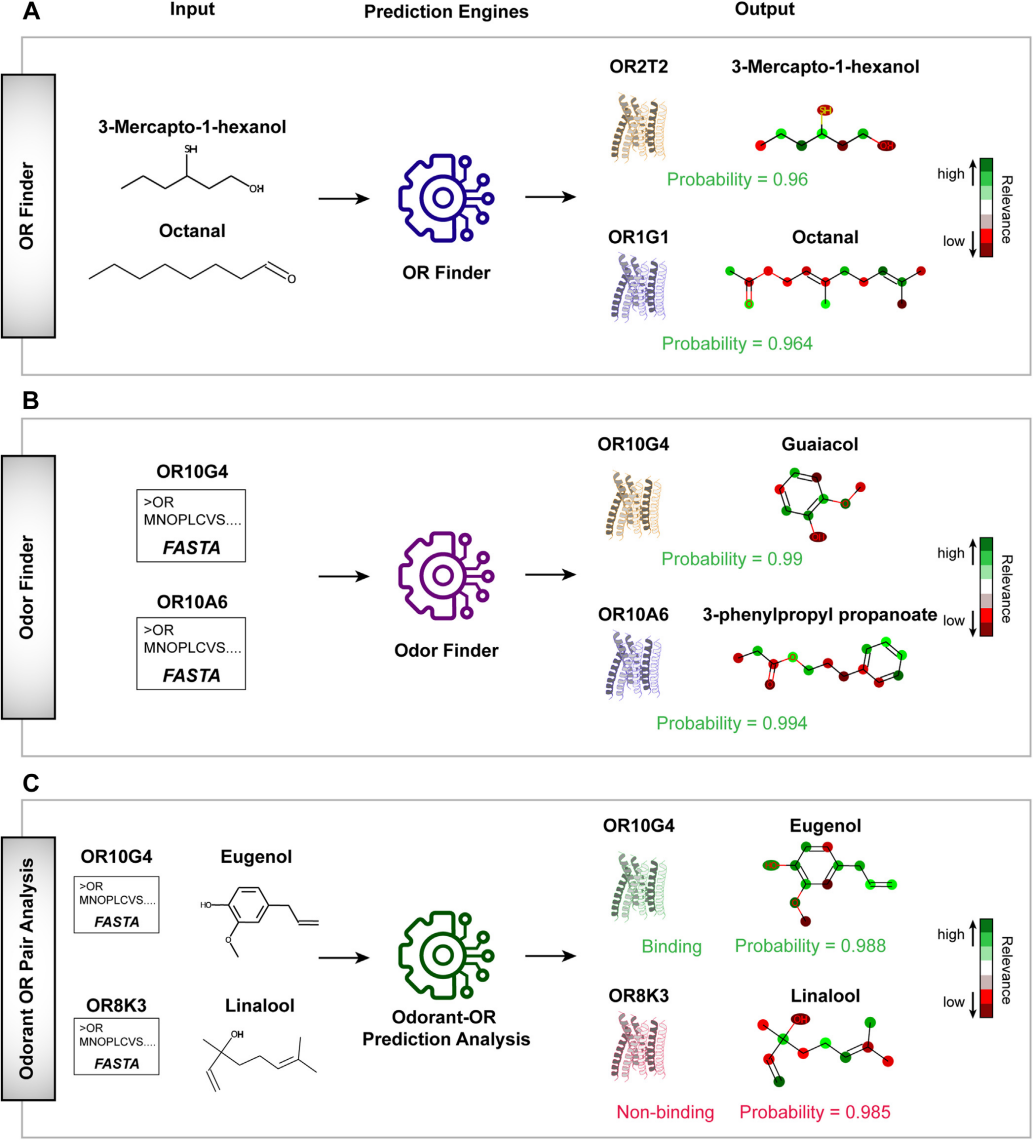
**OdoriFy facilitates multifacet investigation of OR–odorant prediction methods.** Schematic representation depicting the functional workflow of the indicated prediction engines, namely (*A*) OR Finder, (*B*) Odor Finder, (*C*) Odorant–OR Pair Analysis supported by OdoriFy. In all the prediction engines, the user-supplied input passes through the backend supported model, which in turn outputs the prediction results with both the graphical and textual interfaces. In addition to this, it also provides interpretability results both at the atomic (odorant/nonodorant) and amino acid (ORs, if applicable) levels. The *green* and *red colors* represent the contribution of the highlighted atoms toward binding and nonbinding, respectively. Notably, in the case of chemicals, the model first confirms the odorant validation by using Odorant Predictor, and only if it qualifies, then it projects the chemical to the other models for the downstream prediction. OR, odorant receptor.

One of the key distinguishing features of the OdoriFy Web server is that it allows the submission of multiple inputs for prediction and validation of the OR–odorant interactions. In the case of OR Finder, it takes SMILES of the odorant as an input and returns a list of ORs, along with their interaction probabilities (Fig. 4*A* and Fig. S1*B*). Of note, the probability values signify the confidence of interaction between the user-supplied odorant and the predicted ORs. Moreover, it also distinctly highlights the atoms of the input odorant molecule featuring their relevance in the decision making by the model (Fig. 4*A*, Figs. S1*B*, and S4, *A* and *B*). A similar explanation is also provided for the predicted interacting OR, where each amino acid has been provided with a distinct score that quantitatively implies its relative importance in decision making (Figs. S1*B* and S4, *C* and *D*). A similar set of functionalities has been implemented in the Odor Finder prediction engine, but instead of chemicals (SMILES format) as input, it takes OR sequences in the FASTA format (Fig. 4*B* and Fig. S1*B*). Moreover, it returns a list of potential interacting odorant molecules, along with their interaction probabilities. Importantly, similar to the OR Finder, Odor Finder also returns explainability at the atomic level for the odorant molecules (Figs. S1*C* and S5, *A* and *B*) and amino acid level for the ORs (Figs. S1*C* and S5, *C* and *D*). Finally, to further enhance the user experience, OdoriFy also allows users to test whether a given set of OR–odorant pairs interacts or not (Fig. 4*C*, Figs. S1*D*, and S6). This functionality is provided in Odorant–OR Pair Analysis, a separate prediction engine implemented in the OdoriFy Web server. Taken together, OdoriFy harbors a conglomerate of artificial intelligence–driven prediction engines and allows multifacet investigation of odorant–OR interactions.

### OdoriFy synergizes with the structure-based OR–ligand interaction method and outperforms other prediction tools

**Figure 5:**
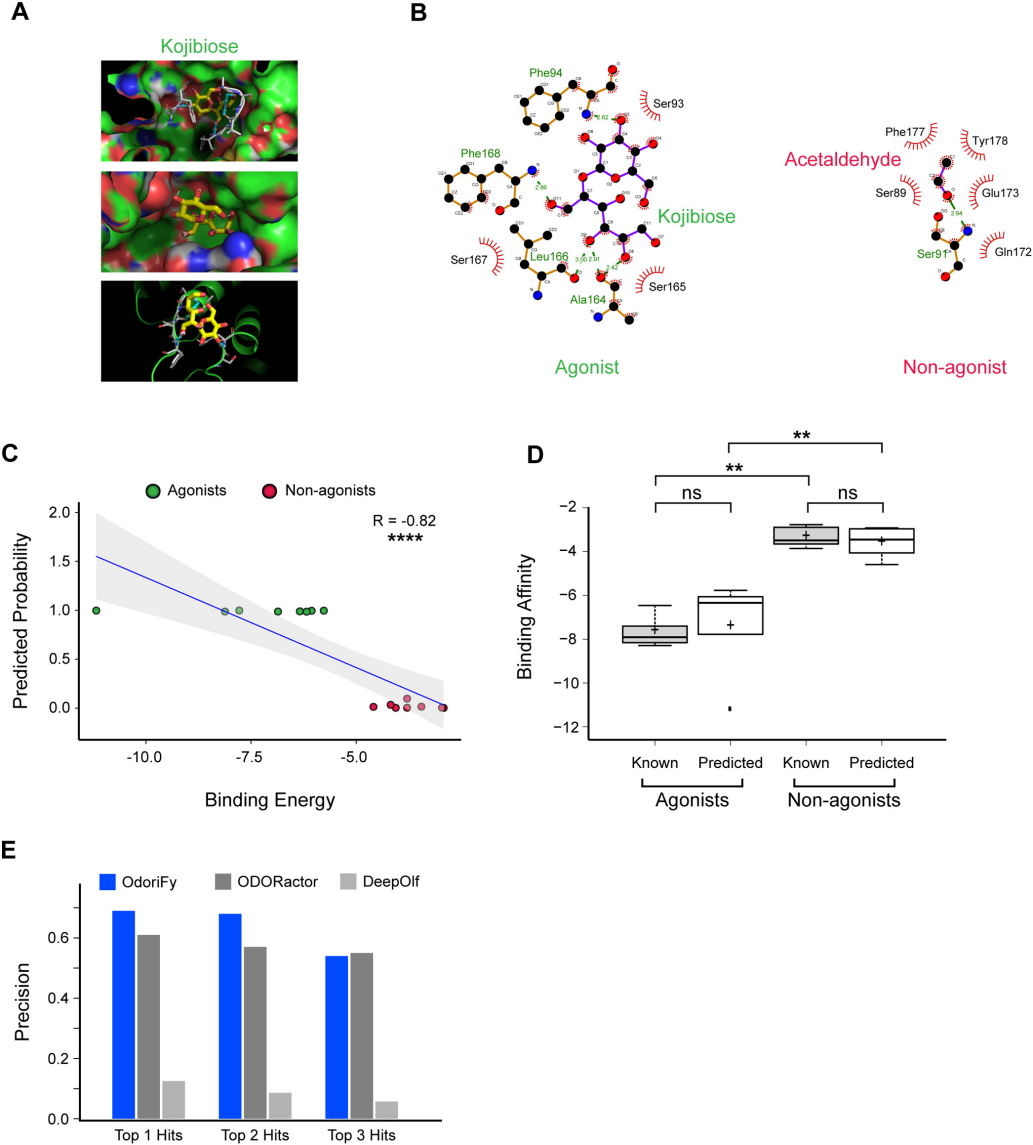
**Crossvalidation of the top predicted agonists/nonagonists of OR1A1 using an orthogonal structure–based approach.***A*, the micrograph depicts the interaction of OR1A1 protein with its agonist Kojibiose inside the binding pocket of a three-dimensional OR1A1 protein structure. *B*, Lig plot highlighting the key amino acids within the binding pocket that are predicted to be interacting with a known agonist Kojibiose (*left*) and nonagonist acetaldehyde (*right*). *C*, scatter plot describing the relationship between predicted OR1A1 agonists (indicated in *green*) and nonagonists (indicated in *red*) with the binding energies obtained using AutoDock. *D*, box plot representing the distribution of the binding energies for the known and predicted agonists and nonagonists. Mann–Whitney *U* test was used for statistical analysis, where ns represents nonsignificant, whereas ∗∗ represents *p* ≤ 0.01. *E*, bar plot representing the comparative performance of OdoriFy, ODORactor, and DeepOlf in precisely predicting the top n hits for the input SMILES/ligands. SMILES, Simplified Molecular-Input Line-Entry System.

An alternative to the prediction-based methods for OR–odorant interaction is the conventional protein structure–based ligand interaction analysis by molecular docking. To date, there is no experimentally resolved structure of any mammalian OR present in the Protein Data Bank (62). As an alternative approach, modeling by the fold recognition method, followed by the molecular dynamics simulation, is a widely adopted technique for the prediction of stable OR structures, and once obtained, dynamic docking is performed between the computationally predicted OR structures and putative odorant molecules. To test whether the OdoriFy predicted values for odorant–OR interactions are meaningful, we performed a comparative analysis with one of the broadly tuned human ORs, OR1A1. Recently, we built a stable structure of OR1A1 (41) using the fold recognition method using GPCR-I-TASSER and further refined it using the molecular dynamics approach (GROMACS, University of Groningen Royal Institute of Technology Uppsala University (63)) (Fig. S8). To crossverify OdoriFy predictions, we selected eight top (high probability) and bottom (low probability) predicted agonists and nonagonists, respectively, and performed dynamic docking (64) within the predicted active site of OR1A1 protein (Fig. 5, *A* and *B*). For the positive and negative controls, we took five known agonists and nonagonists of OR1A1. Our results highlight a significant inverse correlation between binding energies (obtained using docking) and predictive interaction probabilities (obtained using OdoriFy) of agonists and nonagonists, suggesting higher degree of synergy between these two alternative methods (Fig. 5*C*). Of note, we have not observed any significant (*p* > 0.05; Mann–Whitney *U* test) differences in the binding energies of OR1A1 among the known/predicted agonists or nonagonists indicating the robustness of the structure-based method (Fig. 5*D*). We next asked whether OdoriFy prediction models perform competitively as compared with the existing tools. To achieve this, we utilized an independent, held-out validation dataset from our curated interactions. We tested this validation dataset as an input query on two other prediction models, that is, ODORactor (65) and DeepOlf (66). Of note, the comparative analysis was only feasible for computing the model precision because of the functional limitations associated with DeepOlf (66) and ODORactor (65) (Fig. S7). Nevertheless, our analysis revealed that OdoriFy outperformed both these alternative methods under stringent conditions (Top-K hits with K ≤ 2). However, it showed comparable results with ODORactor (65) with lesser stringent conditions (allowing top three hits) (Fig. 5*E*).

## Discussion

In this work, we introduce OdoriFy, a comprehensive Web server equipped with four different prediction engines, that is, Odorant Predictor, Odor Finder, OR Finder, and Odorant–OR Pair Analysis. These four prediction engines collectively allow validation of odorant status, prediction of responsive ORs for given odorants, prediction of putative odorants for user-supplied ORs, and verification of OR–odorant pair interaction. Importantly, all the aforementioned prediction engines also support modules of explainable artificial intelligence, allowing the user to decode the underlying features for the model classification/prediction at the atomic (odorant or nonodorant) and amino acid levels (ORs). Of note, machine/deep learning–based approaches have been used in the past to build prediction models for OR–odorant interactions as well as for the odorant/nonodorant classification (65, 66). Notably, both ODORactor (65) as well as recently introduced DeepOlf (66) utilizes manually designed molecular descriptors for model building as well as for predictions, whereas, OdoriFy models utilize One-Hot encoding/BiLSTM-based techniques. Moreover, the implementation of explainable artificial intelligence components is also exclusive to OdoriFy. Content-rich interface and a high degree of customizability are among the key features of OdoriFy. For instance, OR Finder supports both the normal (slower but more accurate) and rapid (faster but less accurate) testing of user-supplied odorants. The Odor Finder module supports user-defined K values (top K interacting odorants for the query OR sequence), whereas this parameter is fixed in the case of ODORactor (65) and requires changes in the source code of DeepOlf (66). OdoriFy is a secure SSL-certified Web server and supports batch search and returns results *via* electronic mail. Of note, in addition to the aforementioned advancements, OdoriFy is comparably more accurate since the models are trained on large amounts of ground truth data using state-of-the-art deep learning techniques. Despite possessing multiple advantageous features, OdoriFy has certain technical limitations. First, unlike ODORactor (65) and DeepOlf (66), OdoriFy does not support the chemical exploration of the mouse olfactory system since it is exclusively built on human data. Second, OdoriFy utilizes RDKit (http://www.rdkit.org/RDKit_Overview.pdf) to parse and convert SMILES and therefore does not support SMILES containing ionic bonds with a period (.). Finally, OdoriFy does not support input SMILES with multiple letter symbols such as (Br) for Bromine.

We believe that OdoriFy will provide the much-required computational framework in the field of chemosensory research to accelerate the ongoing efforts for ORs deorphanization. It provides a virtual means to prioritize odorants for the *in vivo/in vitro* interaction assays, as opposed to the hit and trial methods. Moreover, OdoriFy functionalities also allow decoding of the molecular basis of odorant–OR interactions, that is, to link the OR amino acid motifs to that of odorant substructures, which has been a challenging task to date. These results provide chemosensory researchers with the key amino acids of the OR protein sequence that contribute to the ligand–receptor interactions and therefore assist the mutagenesis experiments for the *in vitro/in vivo* validations. The odorant/nonodorant dataset that laid the foundation of the Odorant Predictor engine could be utilized to demarcate the boundaries of odorant space, that is, to determine the total number of odorants that humans can detect and discriminate. Importantly, this dataset could also be used to identify the set of key physicochemical properties of the odorants using feature selection/reduction techniques, which could further enhance our basic understanding of odorant chemistry and therefore foster the synthesis of new synthetic odorants (47). OdoriFy server and its associated datasets can be used to explore the relationship between odorant structure and its perception. Earlier studies linked odorant perceptual dimensions to odorant structural dimensions (46, 67, 68). Of note, OdoriFy lacks perceptual information, but the publicly available perceptual datasets could be easily overlaid leading to the multidimensional information-linking odorants with their cognate receptors and perceptual properties (69). In addition to this, OdoriFy can be used to shed light on the evolutionary aspects of olfaction. The majority of the human ORs are pseudogenes and therefore do not directly contribute to odorant detection (20, 70). Odor Finder engine can be used to predict the potential odorants for these pseudogenes. Notably, such an approach requires other computational methods to convert pseudo OR genes into computationally corrected and evolutionary-directed functional genes. An emerging frontier in olfaction is that the overall odor perception is not only triggered by an odorant binding to its cognate receptor but also involves parallel inhibition of other ORs by the same odorant, thereby reinforcing OR inhibition as a fundamental phenomenon to odor encoding (71, 72). OdoriFy harbors the largest compendium of known OR–odorant interactions. Such a large volume of data could be used to dissect the molecular basis of OR inhibition using the heterologous expression–based assays or computer-aided OR–odorant interactions. Moreover, OdoriFy could also contribute to the identification of endogenous ligands for the extranasal ORs. A recent report linked the prostate cancer–associated OR, that is, OR51E2 with the testosterone metabolite 19-hydroxyandrostenedione (73). This seminal work suggests that the extranasal ORs could be potentially activated by the endogenous metabolites. OR Finder prediction engine harbors functionalities to link the extranasal ORs to that of user-provided metabolites (73). Last and most importantly, the underlying deep learning frameworks of OdoriFy could be translated into other subdomains of chemoinformatics research such as *in silico* drug design (74).

## Experimental procedures

### Deep neural networks in chemoinformatics

Machine learning, a subset of artificial intelligence, allows extraction of meaningful patterns or inferences from the provided dataset (termed as training data) and therefore permits the execution of complex tasks like classification or prediction on the unseen data (testing dataset). Deep learning techniques are an efficient and latest subfield of machine learning. A deep neural network is the hierarchical (layered) organization of neurons that are interconnected to other neurons. The transfer of information or signals occurs between neurons and therefore leads to a complex network that learns with feedback mechanisms. The usage of deep learning–based methods has been a recent phenomenon in chemoinformatics (75, 76). The present work utilizes novel deep learning architectures in olfaction and therefore allows classification of chemicals into odorants and nonodorants and identification of putative cognate ORs for the query odorants or *vice versa*.

### Data compilation

The training data for four different OdoriFy prediction engines, namely Odor Finder, OR Finder, Odorant Predictor, and Odorant–OR Pair Analysis were manually compiled from published studies till March 2020, retrieved by PubMed search. Since the performance of machine learning–based classification/prediction models largely depends on the input dataset used for the model training, we followed stringent multiphase steps to ensure the data quality. For this, the initial data compilation and its crossvalidation were performed in three phases. In the first phase, all articles citing interaction information relating to ORs, alongside the information about their known agonists and nonagonists, were downloaded from PubMed, and each of the filtered publications was thoroughly read and the details of the interactions were manually extracted and compiled. In the second phase, the information was reverified and filtered for contradicting entries by a separate team. In the last phase, all the interactions were manually verified by all the authors. A total of 679 agonist-OR pairs and 5474 nonagonist-OR pairs were collated.

Similar data sanity checks were carried out for the dataset used for the Odorant Predictor classification model, which allows verification of odorant properties in user-supplied chemicals. To obtain the training and testing datasets, we compiled the bonafide odorants and nonodorant molecules from two independent repositories, namely PubChem (https://pubchem.ncbi.nlm.nih.gov/) and “thegoodscentscompany” (http://www.thegoodscentscompany.com/). To ensure its validity, we followed all the aforementioned sanity checks. Initial data filtering steps were performed to remove the redundant and conflicting entries. Finally, we obtained a total of 5003 odorants and 857 nonodorants that we subsequently used for training the deep learning model. For describing these chemical moieties in the form of a line notation, we used a Simplified Molecular-Input Line-Entry System (SMILES) representation. Furthermore, to avoid discrepancies between various SMILES subformats, we used the OPSIN tool (56) (https://opsin.ch.cam.ac.uk/) for the uniform conversion of chemicals into SMILES.

### Prediction engines

The graphical user interface of OdoriFy supports four classification/prediction tasks, ranging from the validation of odorant properties in query chemicals to the identification of their cognate ORs.

#### Odorant Predictor

The architecture of the Odorant Predictor module is based on Transformer-CNN (57). Transformer-CNN with the default settings was used for the model generation. The input data were formatted as per the authors' recommendations. One of the key features of Transformer-CNN is that it allows augmentation of the SMILES for training and inference, thereby enabling the construction of robust models even with smaller datasets. In the case of Odorant Predictor, the model was built using the quantitative structure–activity (property) relationship workflow, an inbuilt function in Transformer-CNN (57). The interpretability of the model at the substructure level was performed using the layerwise relevance propagation method (77). Layerwise relevance propagation method is an explanation algorithm used in tandem with neural networks to evaluate and allow the propagation of relevance from the last layer to the input layer. With this approach, the most relevant features can be identified. To build and test the accuracy of the prediction model and avoid training data biases, we performed an iterative process (100 times), in which each cycle involves the random splitting of total data into training (80%) and testing (20%) sets, followed by model building and its performance evaluation. Of note, the performance of each individual model was computed using the standard parameters such as accuracy, balanced accuracy, Cohen's kappa score, precision, recall, the area under the curve of receiver operating characteristic (AUC-ROC), and F1 score. Accuracy is described as the proportion of correct predictions by the model. However, accuracy alone as a measure of model performance may be misleading when exposed to an unequal number of samples in each class, a problem commonly regarded as the class imbalance problem. These limitations can be overcome by the use of balanced metrics that control for class imbalance. Precision provides information on the proportion of positive predictions that are true positives, whereas the recall parameter provides information on the proportion of the true positives that are correctly predicted. F1 score is the harmonic mean of precision and recall. The AUC-ROC provides information about how well the model can distinguish between the classes. The higher the value of AUC-ROC (toward 1), the higher is the capability of the underlying model to distinguish between the classes. Cohen's kappa score compares the accuracy of the model while controlling for the hypothetical scenario of random classification.

#### Odor Finder, OR Finder, and Odorant–OR Pair Analysis

The model underpinning Odor Finder, OR Finder, and Odorant–OR Pair Analysis was built using PyTorch, a python-based deep learning toolkit (78). Of note, a single model was used at the back end that supports each of these prediction engines. The input datasets used for model building constitute protein sequences associated with the ORs, SMILES of their agonists/nonagonists, and the resultant activation status (1 = activation; 0 = nonactivation). For building and testing the efficacy of the model, we followed a similar strategy of iteration and model performance estimation as discussed previously for the Odorant Prediction model. Notably, the input dataset was first filtered through Odorant Predictor to remove any nonodorants from the data. Importantly, the majority of the agonists-OR pairs (643 of 679) and nonagonists-OR pairs (5390 of 5474) in the dataset qualified for odorant properties (prediction probability cutoff > 0.5) and therefore were used for the downstream model building. In the case of the Odorant–OR Pair Analysis prediction engine, the input data were randomly split into training (80%) and testing (20%) datasets. The former dataset was used for model building, whereas the latter was used for the performance estimation. Notably, the odorant–OR interaction data contained a higher number of nonagonists, as compared with agonists. To circumvent this, we oversampled the minority class using Resample from the *sklearn* python package to ensure equal contribution from both classes. The OR sequences and their respective agonists and nonagonists SMILES from both the datasets were One-Hot encoded (conversion of a sequence of symbols into bit vectors) using the following strategy. One-Hot encoding of the receptor sequence was done by taking 26 capital letters and one special character (ABCDEFGHIJKLMNOPQRSTUVWXYZˆ), and One-Hot encoding of SMILES was done for the following 77 characters:

()+–./−0123456789=#@$ABCDEFGHIJKLMNOPQRSTUVWXYZ[∖]abcdefghijklmnopqrstuvwxyzˆ

Two independent Bidirectional Long Short-Term Memory (BiLSTM) networks were applied on the One-Hot encoded vectors of SMILES and receptor sequence, respectively. Both these individual layers were then passed through the linear layers of the network, and their resulting vectors were concatenated. This was followed by hidden layers with rectified linear unit activation functions and Softmax as the output layer. We used dropout to prevent model overfitting. Hyperparameter tuning using grid search revealed the optimal lengths for SMILES and OR, which are 75 and 315, respectively. For both SMILES and OR sequences, 50 turned out to be the optimal number for hidden layers. All our model performance measurements were carried out on bootstrapped training and validation datasets, generated by random sampling.

OR finder identifies the potential responsive ORs for the query odorant/SMILES. For this, instead of searching the entire dataset to fetch the user-supplied SMILES, and return their cognate receptors, we narrowed our search space by applying this search function on a subset of dataset SMILES, which are structurally similar SMILES to the user query. This was achieved using the Tanimoto similarity metric for fingerprinting and computing the molecular similarity using RDKit (http://www.rdkit.org/RDKit_Overview.pdf) (https://github.com/rdkit/rdkit). After obtaining the limited set of OR sequences, we calculated their binding probabilities with the query SMILES by BiLSTM-based model (discussed previously). In addition to this approach, we also employed a brute force approach wherein we predicted the binding nature of the query SMILES with each unique receptor sequence available in our manually curated database. This step checks the potential binding of the input SMILES with all human ORs and returns the top K ORs (10 ≥ K ≥ 1), along with the information about their protein sequences and binding probabilities. It is undoubtedly slower but theoretically more accurate and more comprehensive than similarity-based prediction methodology (discussed previously) (79, 80). Of note, the prediction model was trained on both the mutant and wildtype ORs; however, in the case of OR Finder, the prediction engine only checks for binding with wildtype receptors (*i.e.*, ~400 unique human ORs). The output of the OR finder provides a list of potential ORs along with their binding probabilities for the user-provided odorants.

In the Odor Finder prediction engine, which enables the identification of odorant molecules for wildtype or mutant human ORs, we used the aforementioned brute force approach for scanning our database. Of note, the underlying model used here is the same as the one we used for the Odorant–OR Pair Analysis. Outputs of each prediction engine are then converted into comma-separated values (CSVs) and displayed to users. We implemented an interpretability module for each prediction engine to gain insights into the “why” of a prediction. Our interpretability module returns the positively and negatively contributing atoms with shades of green and red, respectively. The interpretability module is built on the integrated gradients, which provide a visual representation of the importance of the input features contributing to the model's prediction (Captum: https://github.com/pytorch/captum) (58, 78). Such approaches have been extensively used in the drug discovery domain, sentiment analysis, natural language processing, image classifications, and so on. For substructure analysis and highlighting atoms, we used the RDkit Python library (http://www.rdkit.org/RDKit_Overview.pdf) that draws molecules and colors them individually according to their relevance in the model's predictions.

### Implementation of the frontend

OdoriFy is built on the open-source cascading style sheets framework, utilizing the widely popular bootstrap with Popper.js, and jQuery (https://jquery.com/), javascript library. Apart from providing an overall theme to the Web server, advanced techniques such as lazy loading of style sheets/images and rendering minified HTML have been used to ensure fast loading times. Given an anticipated load of requests on the Web server and possible security threats, instead of processing an uploaded CSV on the server side, the parsing happens on the client side *via* Papa Parse.js (https://github.com/mholt/PapaParse). As a CSV is uploaded, each row of the CSV is processed using papaparse.js, based on the prediction engine. For Odorant Predictor and OR Finder, the desired CSV consisting of a single column with SMILES in each row is required. For Odor Finder, the desired CSV consists of two columns of FASTA rows, the first column being the FASTA header and the other column, the receptor sequence. Odorant–OR Pair Analysis on the other hand requires an additional column of SMILES in its input CSV file. For populating the results on the frontend and achieving maximum possible modularity while creating the views, Django templating has been used to render the pages with the context being received from the Django controller (version 3.1.5; https://www.djangoproject.com/). The contact form parameters are received using a get request and then using simple mail transfer protocol, an acknowledgment mail is sent to the user assuring that their query will be processed as soon as possible.

## Data availability

OdoriFy is an open-source tool with multiple prediction engines. It is provided as a Web server (https://odorify.ahujalab.iiitd.edu.in). Moreover, the source code of OdoriFy can be obtained from https://github.com/the-ahuja-lab/Odorify-webserver. The source code of the embeddings needed to train the model is available at https://github.com/the-ahuja-lab/Odorify.

## Supporting information

This article contains supporting information.

## Conflict of interest

The authors declare that they have no conflicts of interest with the contents of this article.
