# Supplementary material for: OdoriFy: A conglomerate of artificial intelligence–driven prediction engines for olfactory decoding
Source: J Biol Chem. 2021 Jul 12;297(2):100956. doi: 10.1016/j.jbc.2021.100956 (PMC8342790; doi:10.1016/j.jbc.2021.100956)
Supplement: Supplemental Figures S1–S8 [file mmc1.pdf]

OdoriFy: A conglomerate of Artificial Intelligence-driven prediction engines for olfactory decoding

Ria Gupta[†,1], Aayushi Mittal[†,1], Vishesh Agrawal[†,1], Sushant Gupta[1], Krishan Gupta[2], Rishi Raj Jain[3], Prakriti Garg[1], Sanjay Kumar Mohanty[1], Riya Sogani[1], Harshit Singh Chhabra[2], Vishakha Gautam[1], Tripti Mishra[6], Debarka Sengupta[1,2,4,5] and Gaurav Ahuja[1]

[1]Department of Computational Biology, Indraprastha Institute of Information Technology-Delhi (IIIT-Delhi), Okhla, Phase III, New Delhi-110020, India
[2]Department of Computer Science and Engineering, Indraprastha Institute of Information Technology-Delhi (IIIT-Delhi), Okhla, Phase III, New Delhi-110020, India
[3]Department of Computer Science and Design, Indraprastha Institute of Information Technology-Delhi (IIIT-Delhi), Okhla, Phase III, New Delhi-110020, India
[4]Centre for Artificial Intelligence, Indraprastha Institute of Information Technology, New Delhi, India.
[5]Institute of Health and Biomedical Innovation, Queensland University of Technology, Brisbane, Queensland, Australia.
[6]Pathfinder Research and Training Foundation, 30/7 and 8, Knowledge Park III, Greater Noida, Uttar Pradesh - 201308, India

[†]Equal contribution

To whom correspondence should be addressed. Gaurav Ahuja; Email: gaurav.ahuja@iiitd.ac.in.

Running title: OdoriFy: An AI-based webserver for human olfaction

Supplementary Information

Supplementary Figure 1: Graphical User Interface of OdoriFy webserver

A snippet of the graphical user interface of OdoriFy supported prediction engines. It also contains information about the required format of the input data for each prediction engine, along with descriptions about the possible outputs from the server.

A

Odorant Predictor

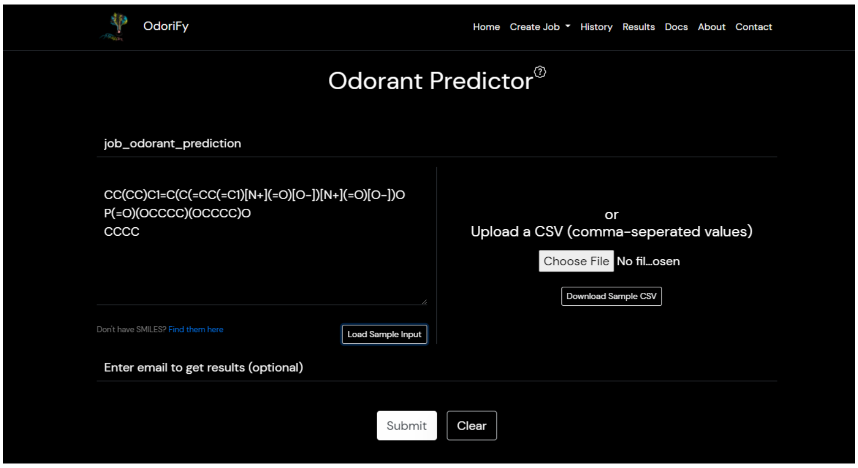

Input: SMILES

Output:

- Odorant or Non-odorant
- Prediction Probability
- SMILES Interpretability (Bar Graph)
- SMILES Interpretability (Structure)

B

OR Finder

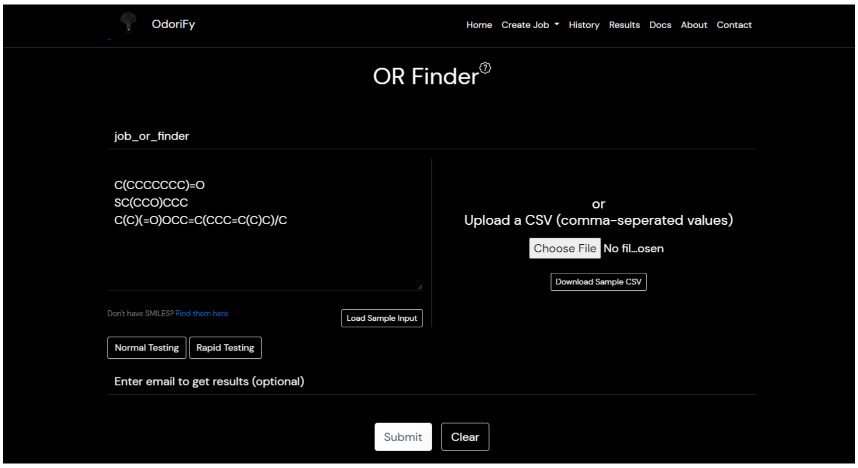

Input: SMILES

Output:

- Receptor Name
- Sequence
- Prediction Probability
- Receptor Sequence Interpretability
- SMILES Interpretability (Bar Graph)
- SMILES Interpretability (Structure)

C

Odor Finder

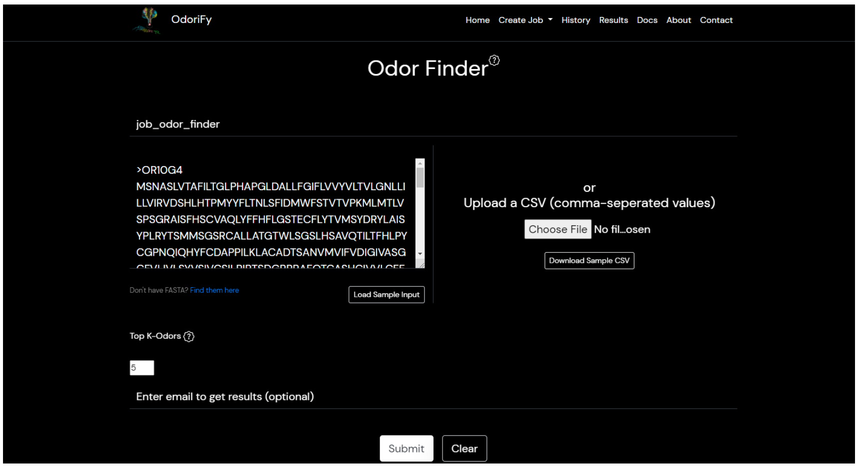

Input: FASTA

Output:

- Odor (SMILES)
- Prediction Probability
- Receptor Sequence Interpretability
- SMILES Interpretability (Bar Graph)
- SMILES Interpretability (Structure)

D

Odorant-OR Pair Analysis

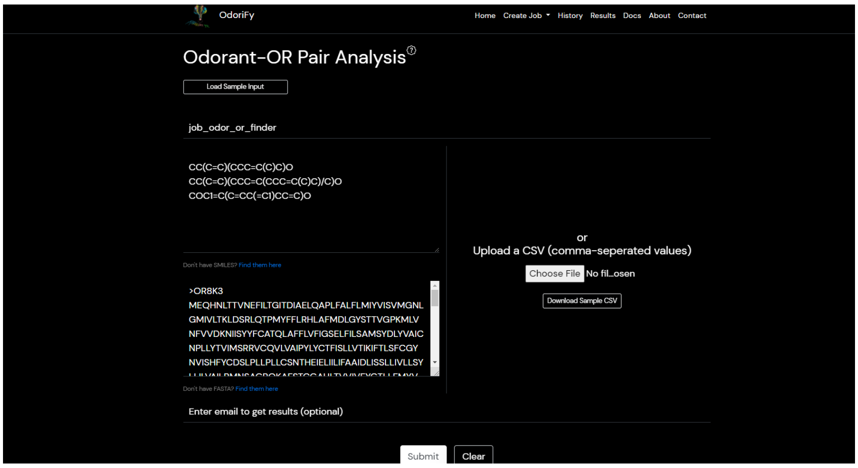

Input: SMILES and FASTA

Output:

- SMILES
- Sequence
- Activation Status
- Prediction Probability
- Receptor Sequence Interpretability
- SMILES Interpretability (Bar Graph)
- SMILES Interpretability (Structure)

Figure S1

Supplementary Figure 2: Odorant Predictor allows model explainability at the atomic level for the odorants or non-odorant molecules.

(A) Box plot representing the distribution of 100 random iterations of the key metrics collectively describing the performance of the model on the testing dataset. The indicated metrics include accuracy, balanced accuracy, ROC-AUC, Cohen's kappa, F1 Score, precision, and recall. **(B)** Bar graph representing the absolute counts of the 12 prominent functional groups enriched in the bonafide odorants and non-odorants, collectively describing the functional group enrichment in the input dataset. This analysis is performed using the ChemmineR package. **(C-D)** Bar graphs indicating the relevance of each element of the input SMILES in model classification decisions in the indicated examples.

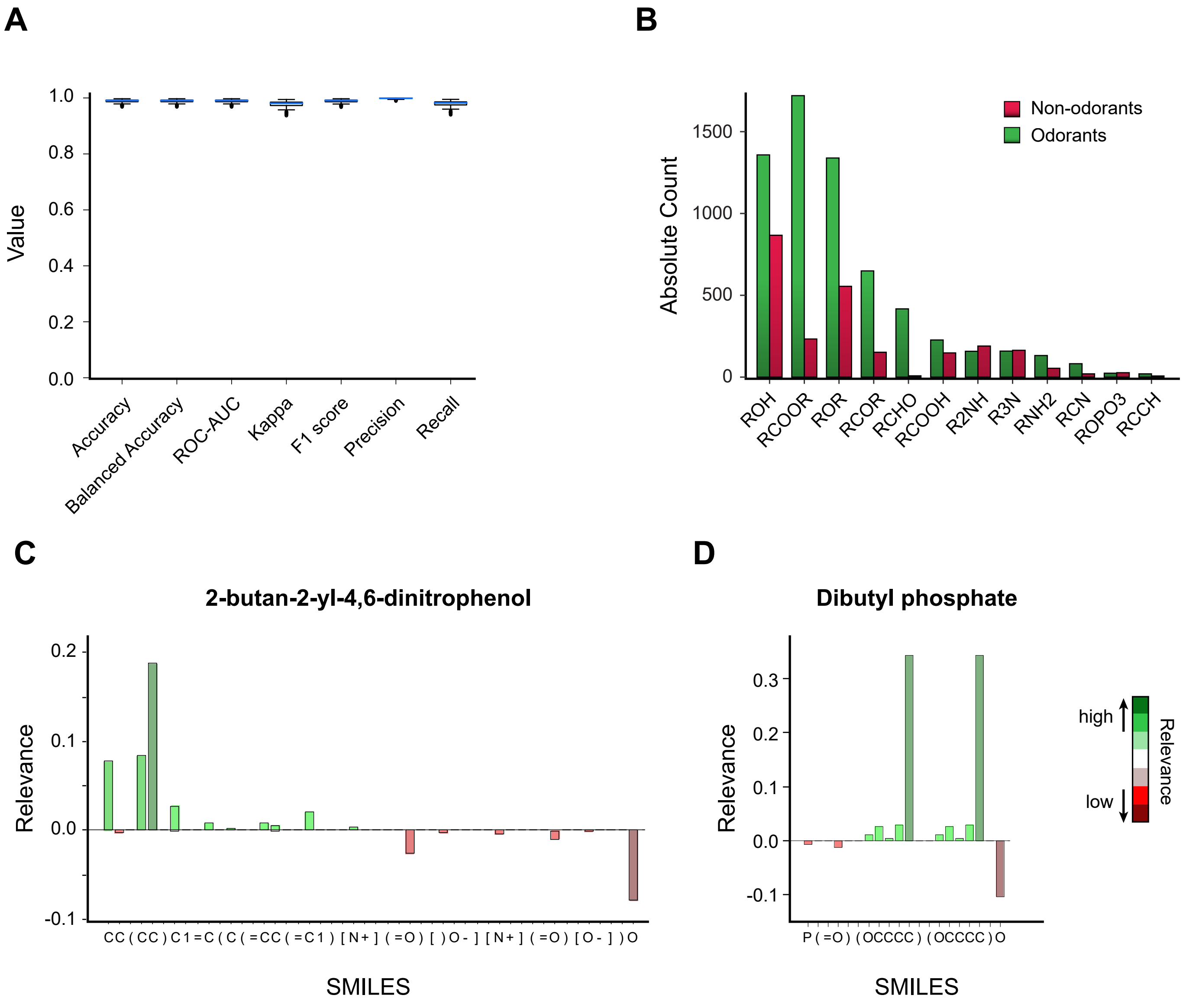
A
Value
1.0
0.8
0.6
0.4
0.2
0.0
Accuracy
Balanced Accuracy
ROC-AUC
Kappa
F1 score
Precision
Recall
B
Absolute Count
1500
1000
500
0
Non-odorants
Odorants
ROH
RCOOR
ROR
RCOR
RCHO
RCOOH
R2NH
R3N
RNH2
RCN
ROPO3
RCCH
C
2-butan-2-yl-4,6-dinitrophenol
Relevance
0.2
0.1
0.0
-0.1
CC(CC)C1=C(C(=CC(=C1)[N+](=O)[)O-][N+](=O)[O-])O
SMILES
D
Dibutyl phosphate
Relevance
0.3
0.2
0.1
0.0
-0.1
P(=O)(OCCCC)(OCCCC)O
SMILES
high
low
Relevance

Figure S2

Supplementary Figure 3: Deep Neural Network-mediated learning of receptor-odorant interactions.

(A) Bar graph depicting the shift in the number of OR-agonists and OR-non-agonist pairs in the initially compiled dataset, before and after filtering via Odorant Predictor. **(B)** The line plot depicting the changes in accuracy over epochs for training (blue) and testing (orange) datasets, respectively. Box plots representing the distribution of 100 random iterations of the key metrics collectively describing the potency of the model for **(C)** training and **(D)** testing datasets. The indicated metrics include model accuracy, balanced accuracy, ROC-AUC, Cohen’s kappa, F1 Score, precision, and recall.

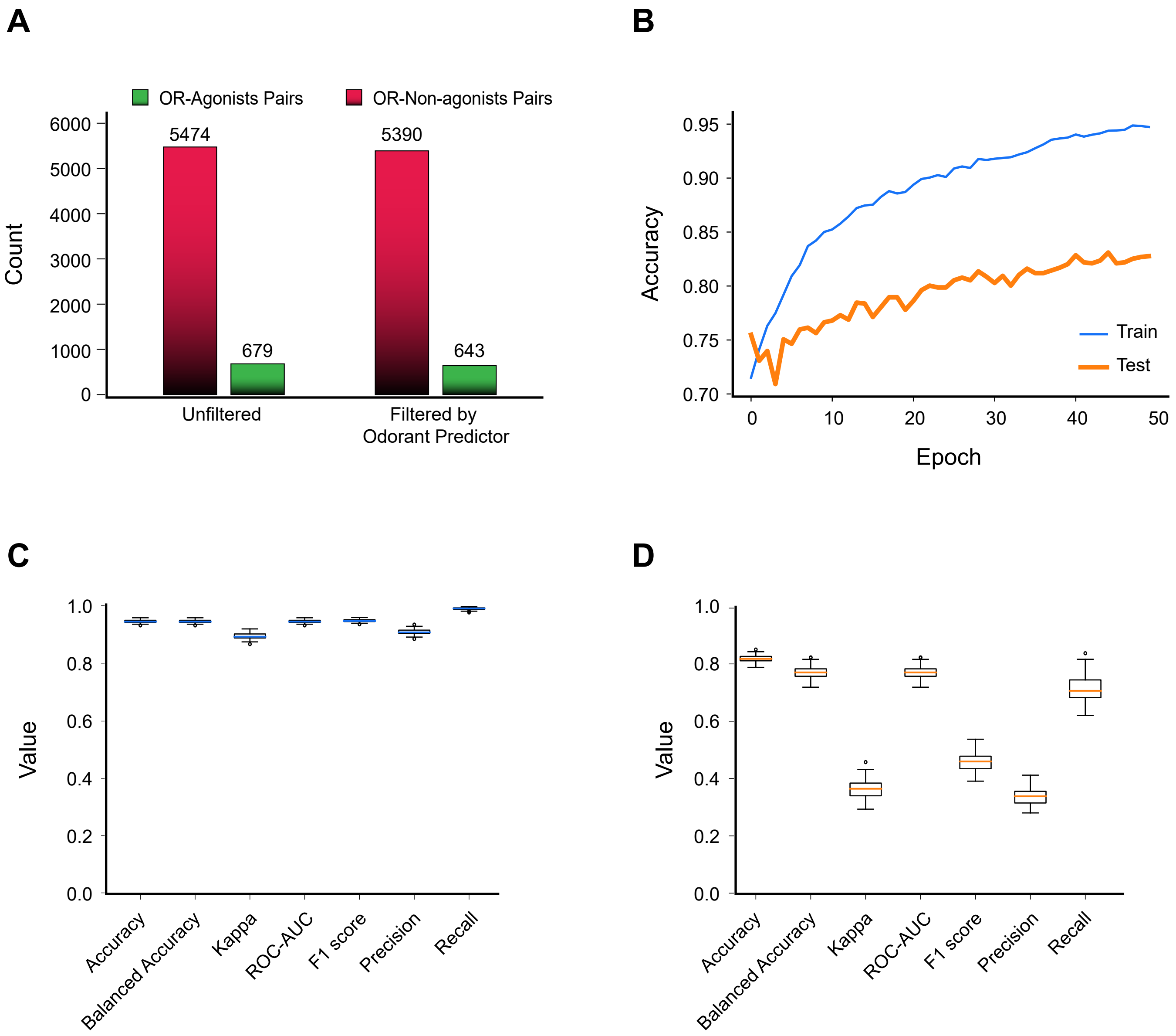
A
B
C
D
OR-Agonists Pairs
OR-Non-agonists Pairs
Count
5474
679
5390
643
Unfiltered
Filtered by Odorant Predictor
Accuracy
Epoch
Train
Test
Value
Accuracy
Balanced Accuracy
Kappa
ROC-AUC
F1 score
Precision
Recall

Figure S3

Supplementary Figure 4: OR Finder allows model explanation at the atomic and amino acid levels

(A-B) Bar graphs indicating the functional relevance of each element of the input SMILES with the predicted receptors. **(C-D)** Bar graphs depicting the interaction relevance of each amino acid of the predicted OR towards user-supplied odorants.

OR Finder

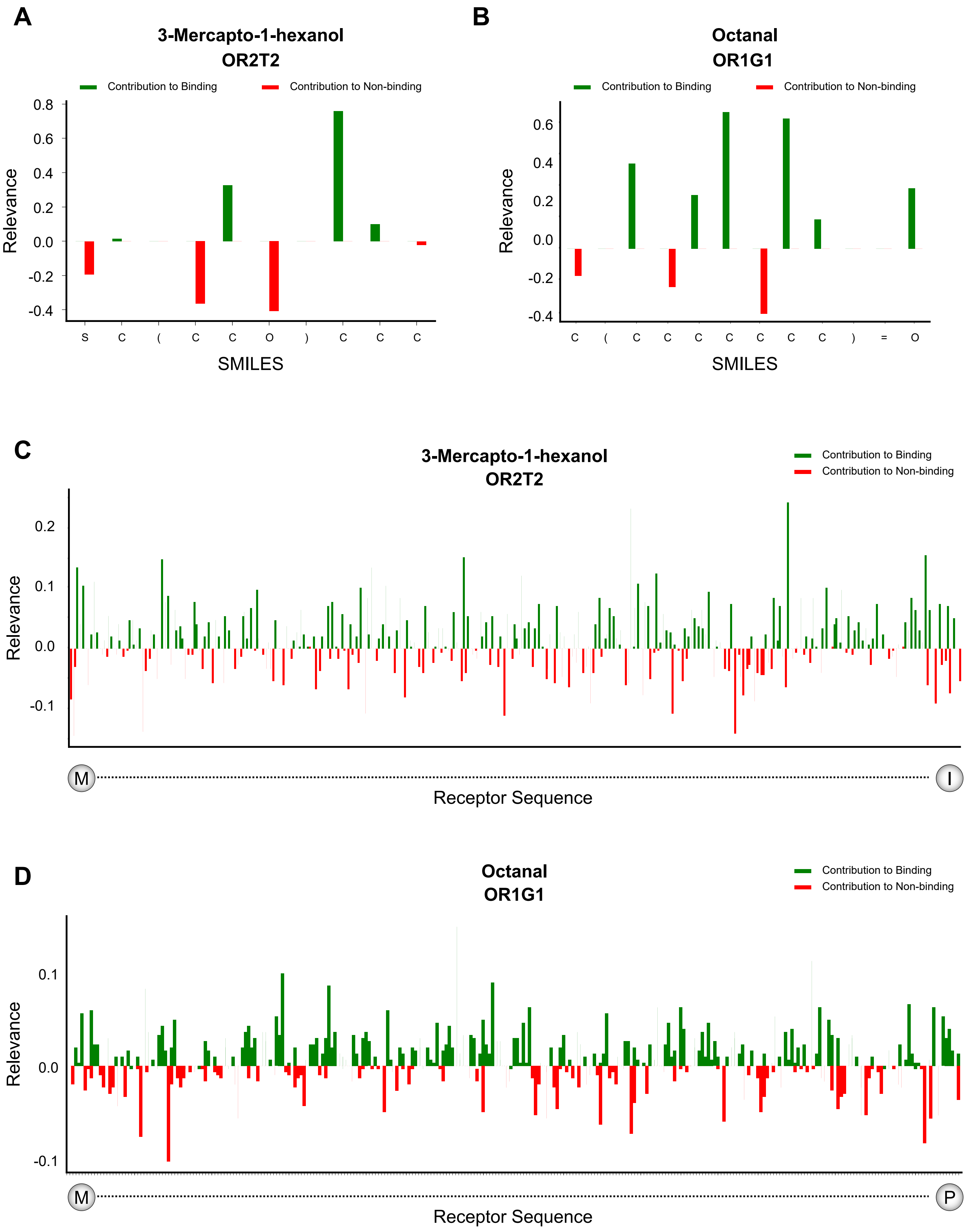

Figure S4

Supplementary Figure 5: Odor Finder allows model explanation at the atomic and amino acid levels

(A-B) Bar graphs depicting the relevance of each element of the predicted chemical towards binding (green) or a non-binding (red) with the user-supplied input receptors. **(C-D)** Bar graphs indicating the interaction relevance of the input OR at the amino acid level towards binding (green) or non-binding (red) with the predicted odorants.

Odor Finder

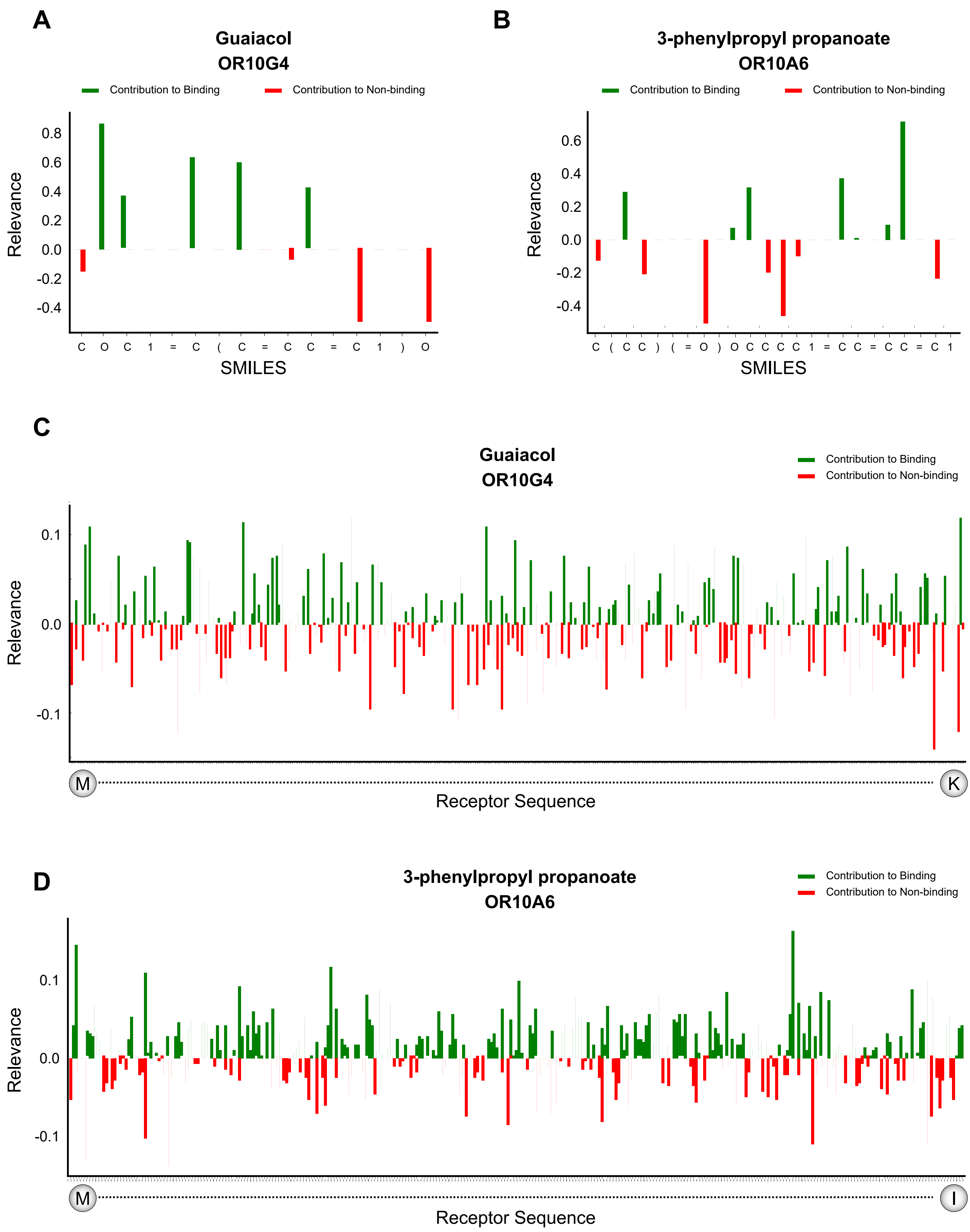

Figure S5

Supplementary Figure 6: Interpretability of the predictions of Odorant-OR Pair Analysis

(A-B) Bar graphs depicting the relevance of each element of the input chemical towards binding (green) or a non-binding (red) with the input receptors. Eugenol with the receptor OR10G4, and Linalool with the Receptor OR8K3. **(C-D)** Bar Graph depicting the relevance of each amino acid of the input OR towards binding (green) or non-binding (red) with the input odorants.

Odorant-OR Pair Analysis

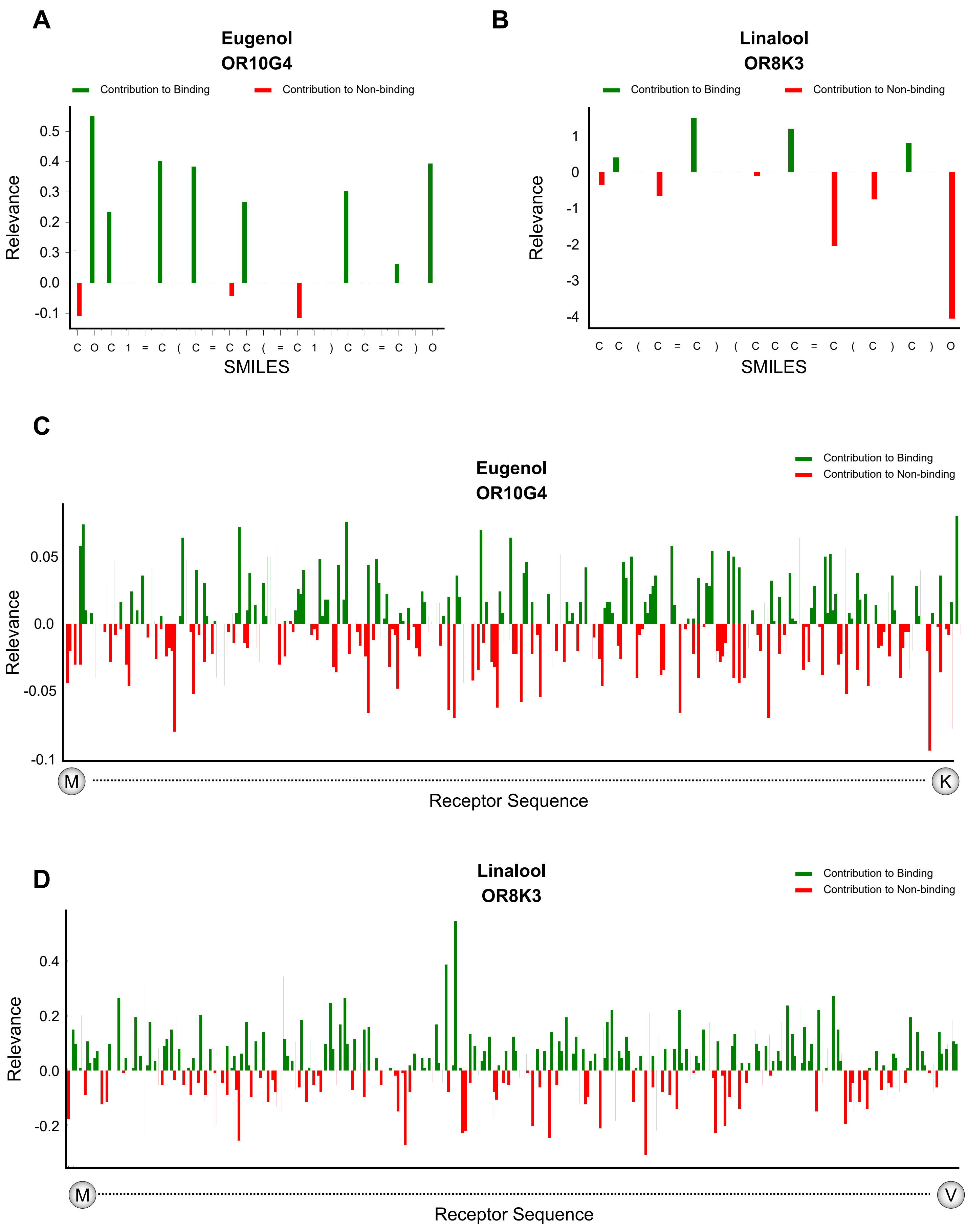

Figure S6

Supplementary Figure 7: Comparative analysis of the key features supported by OdoriFy, ODORactor, and DeepOlf.

Tabular representation highlighting the key differences and similarities between OdoriFy and ODORactor and DeepOlf.

Feature	OdoriFy	DeepOlf	ODORactor
**Odorant Predictor**	Works	Works	Works
**OR Finder**	Works	Works	Works
**Odor Finder**	Works	Doesn't work	Doesn't work
**Odorant-OR Pair Analysis**	Works	Doesn't work	Doesn't work
**Interpretability**	Works (provides bar graph and substructure representations)	Doesn't work	Doesn't work
**Molecular Descriptors**	Not needed	Needed	Not needed
**Value of K (number of output receptors**	Can be modified	Can be modified in source code	Cannot be modified
**Web Server**	Yes	No	Yes
**Similarity Scoring (Rapid Testing)**	Yes	No	No
**Batch Mode**	Yes (25 max)	Not in default code	No
**User Interface**	User Friendly	Difficult for non-coders	User Friendly
**Input Format**	SMILES and/or FASTA can be copy-pasted, or upload a CSV	Molecular Descriptors of SMILES in CSV format only	SMILES, MDL file, MOL file, CAS ID, Draw Structure, Cannot upload a CSV
**Prediction Probability**	Yes	Yes	Yes
**Organisms**	Human	*Homo sapien* and *Mus musculus,* cannot select any one	Human and *Mus musculus*, can select any one
**Run Time**	Different for different prediction engines	Very long	A few seconds, depending on the complexity of input molecule
**Output**	Returns OR names and its protein sequence	Returns OR names only	Returns OR names only
**Details of Molecule and Receptor Probability Graphs**	No	No	Yes
**Downloadable Results**	Yes (zip file containing all graphs and table in CSV format)	Output in CSV format	Very poor format of downloaded results
**Webserver Functionality Checks**			
**Multiple Tabs**	No	Not a Web server	Yes
**Security**	Secure SSL certificates added	Not a Web server	Not secure
**User Login**	Not required	Not a Web server	Not required
**Email Results Functionality**	Yes	Not a Web server	No

Figure S7

Supplementary Figure 8: Molecular Dynamics-based refinement of OR1A1 protein structure.

(A) Ramachandran plots depicting the location of the amino acids of the OR1A1 modeled protein in the favored, allowed, and disallowed regions after simulation refinements. **(B)** Bar plot depicting the error rate of the prediction for each residue of the OR1A1 modeled protein. Almost 80% of the residues fall below the 95% and 99% rejection line and thus making this model a good representative of the stable structure of OR1A1. The two lines on the error axis, 95%, and 99% represent the confidence with which it is possible to reject regions that exceed that error value.

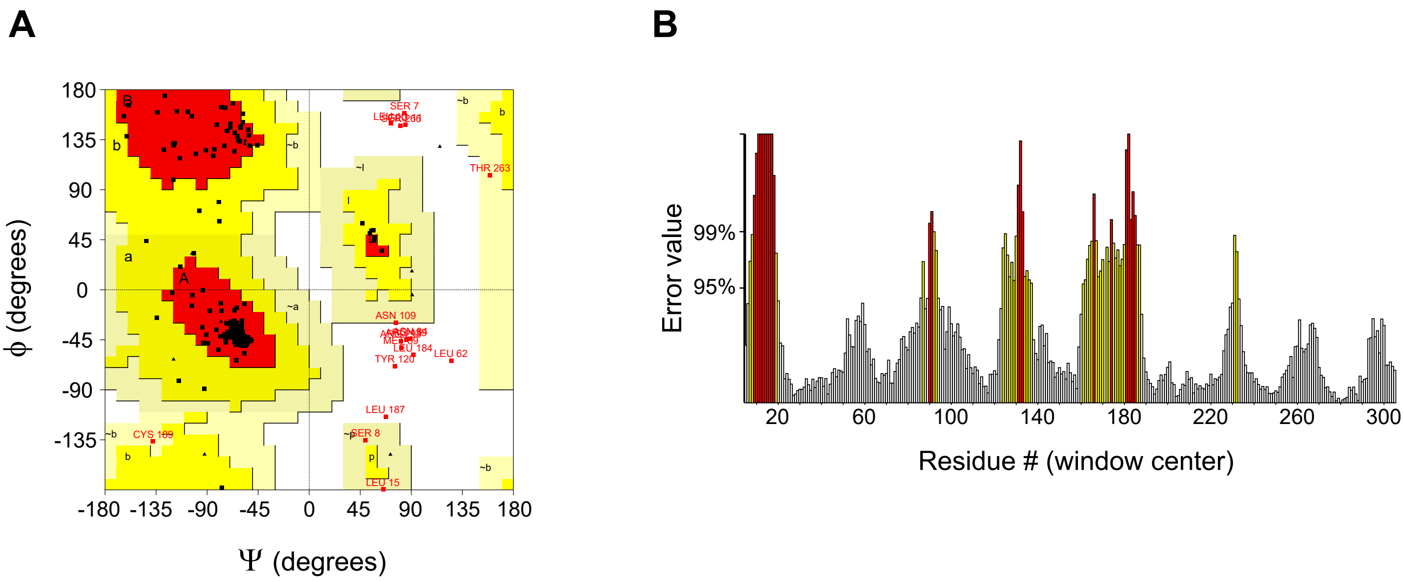
A
B
180
135
90
45
0
-45
-90
-135
ϕ (degrees)
-180 -135 -90 -45 0 45 90 135 180
Ψ (degrees)
SER 7
THR 263
ASN 109
LEU 62
TYR 120
LEU 187
CYS 169
SER 8
LEU 15
99%
95%
Error value
20 60 100 140 180 220 260 300
Residue # (window center)

Figure S8

TABLE LEGENDS

Supplementary Table 1: Tabular representation of the input dataset containing information about bonafide odorants (marked as 1) and non-odorants (marked as 0) along with their SMILES, CAS Registry Numbers, and source from which the information has been extracted.

Supplementary Table 2: Table listing the PMIDs of the publications from which OR-agonist or OR-non-agonist paired data have been extracted.
